# Urinary exosome miR-146a is a potential marker of albuminuria in essential hypertension

**DOI:** 10.1186/s12967-018-1604-6

**Published:** 2018-08-14

**Authors:** Javier Perez-Hernandez, Dolores Olivares, Maria J. Forner, Ana Ortega, Elena Solaz, Fernando Martinez, Felipe J. Chaves, Josep Redon, Raquel Cortes

**Affiliations:** 1 Cardiometabolic and Renal Risk Research Group, INCLIVA Biomedical Research Institute, Avd. Menendez Pelayo, accesorio 4, 46010 Valencia, Spain; 2 Genomic and Genetic Diagnosis Unit, INCLIVA Biomedical Research Institute, Valencia, Spain; 3 Internal Medicine Unit, Hospital Clínico Universitario, Valencia, Spain; 4 CIBER of Diabetes and Associated Metabolic Diseases (CIBERDEM), Institute of Health Carlos III, Minister of Health, Barcelona, Spain; 5 CIBER Physiopathology of Obesity and Nutrition (CIBEROBN), Institute of Health Carlos III, Minister of Health, Madrid, Spain

**Keywords:** Exosomes, microRNAs, Albuminuria, Hypertension, Urinary biomarkers

## Abstract

**Background:**

There is increasing interest in using extracellular vesicle-derived microRNAs (miRNAs) as biomarkers in renal dysfunction and injury. Preliminary evidence indicates that miRNAs regulate the progression of glomerular disease. Indeed, exosomes from the renal system have provided novel evidence in the clinical setting of albuminuria. Thus, the aim of this study was to quantify the urinary miRNAs present in exosome and microvesicles (MVs), and to assess their association with the presence of increased urinary albumin excretion in essential hypertension.

**Methods:**

Exosomes were collected from urine specimens from a cohort of hypertensive patients with (n = 24) or without albuminuria (n = 28), and from 20 healthy volunteers as a control group. Urinary exosomes were phenotyped by Western blot, tunable resistive pulse sensing, and electronic microscopy. Expression of miR-146a and miR-335* was analysed by qRT-PCR and any associations between albuminuria and exosomal miRNAs were analysed.

**Results:**

Urinary miRNAs are highly enriched in exosome subpopulations compared to MVs, both in patients with or without increased albuminuria (p < 0.001), but not in the control group. High albuminuria was associated with 2.5-fold less miR-146a in exosomes (p = 0.017), whereas miR-146a levels in MV did not change. In addition, exosome miR-146a levels were inversely associated with albuminuria (r = 0.65, p < 0.0001), and discriminated the presence of urinary albumin excretion presence [area under the curve = 0.80, 95% confidence interval: 0.66–0.95; p = 0.0013].

**Conclusions:**

Our results indicate that miRNAs were enriched in the urinary exosome subpopulation in hypertensive patients and that low miR-146a expression in exosomes was associated with the presence of albuminuria. Thus, urinary exosome miR-146a may be a potentially useful tool for studying early renal injury in hypertension.

## Background

Increased urinary albumin excretion (UAE) is a marker for cardiovascular and renal disease [1–3]. Its prognostic value in hypertensive patients without diabetes and in subgroups of the general population has been well established over the past few years [4–6]. In presence or absence of diabetes, elevated blood pressure is one the main factors related to increased UAE. Moreover, the management of hypertension reduces the levels and the progressive rate of rise of UAE [7]. Even though an association between blood pressure and UAE has been established, the mechanisms mediating albuminuria progression in hypertensive patients are less well-understood. Thus, in recent years, interest in the potential role of epigenetics in the origin and progression of renal lesions in hypertension has increased.

microRNAs (miRNAs) are small (18–22 nucleotides), non-coding RNA molecules which are post-transcriptional regulators of gene expression [8]. The expression of these molecules is often tissue-restricted, and this fact has led to the idea that miRNA expression may have organ-specific roles [9, 10], and that these miRNAs are also delivered to different extracellular biofluids [11]. Exosomes are 40–130 nm lipid bilayer membrane-derived vesicles from endocytic compartments, that have been shown to play a novel role in cell communication and the interchange of intercellular genetic material [12]. Microvesicles are distinctive from exosomes in that they are produced by shedding of the plasma membrane and usually larger in size (150 nm–1 µm). Besides lipids, and proteins, exosomes also contain various nucleic acid species including functional miRNAs and small RNA, but little mRNA [13]. However, it has been suggested that microvesicles hold functional messenger RNA (mRNA) and little miRNA [14].

It is believed that urinary exosomes may be derived from the kidney and urinary tract, and that these are passively filtered through the glomerulus and/or secreted by the renal tubules [12, 15]. Therefore, the urinary exosome cargos could be generated by the glomerular (podocytes, mesangial and endothelial cells) and tubular cells to exert their functions throughout of the nephron. Therefore, exosomes may constitute paracrine effectors in the crosstalk, between different cell types in the kidney [16].

Preliminary evidence indicates that miRNAs may regulate the progression of glomerular and tubular disease. Therefore, selective sorting of miRNAs into exosomes from the renal system may provide novel evidence in the clinical setting of albuminuria [12, 17, 18]. Our group previously showed that urinary exosome miRNAs are enriched in patients with systemic lupus erythematosus and that lupus nephritis (proteinuria) increases the quantity of urinary exosomal miRNAs, especially miR-146a [19]. The miR-146a is a key regulator of immune responses negatively modulating the interferon-γ pathway [20], has been also involved in renal inflammation [9] and fibrosis regulating extracellular matrix protein production in diabetes [21]. In addition, previous works reported that miR-146a was augmented in lupus nephritis [19, 22] and that is highly expressed in podocytes [23, 24]. While, miR-335* is an abundant miRNA in urine to test the specificity of miR-146a association results with UAE [11].

However, whether extracellular vesicle (EV) miRNA expression is different between exosomes and microvesicles, whether EV miRNA levels are increased or can serve as biomarker for early renal injury in hypertension remains largely unknown. In the present study, we investigated the selective sorting of miRNA into exosomes (high levels than in microvesicles) and the potential role of urinary exosome miRNAs as novel markers of early lesion in hypertension.

## Methods

### Subjects

Urinary samples were collected from patients with essential hypertension who were recruited at the Internal Medicine Department in the Hospital Clínico Universitario in Valencia (Spain). Hypertension was defined according the European Society of Hypertension criteria: systolic blood pressure ≥ 140 mmHg and/or diastolic blood pressure ≥ 90 mmHg) [7]. Of these patients 24 had albuminuria (UAE normalized by urinary creatinine > 30 mg/g) and 28 were normoalbuminuric. All patients received antihypertensive treatment at the time the study was carried out, and average time to disease progression was almost 5 years. Twenty healthy volunteers were analysed as a control group with normal renal function, normal urinalysis, and no history of urinary tract infection, renal stones, or other renal or genitourinary disease (age 35.3 ± 7.0, 70% female). The study protocol was approved by the Ethics Committee of the Hospital Clínico Universitario of Valencia in accordance with the Declaration of Helsinki of 1975 as revised in 2008. All subjects have signed a written informed consent.

### Measurement of urinary albumin excretion

Urinary albumin excretion was measured in morning urine collections using a nephelometric immunoassay (Behring Institute). The level of albuminuria for each patient was considered as the mean value obtained from the morning spot urine samples and expressed as the albumin (mg) to creatinine (mg) ratio (ACR). Increased UAE was defined as an ACR ≥ 30 μg/mg. In our laboratory, the intra-assay, inter-assay, and intra-individual coefficients of reproducibility for UAE measurement were 2%, 6%, and 12%, respectively.

### Exosome isolation

The two EV subpopulations—exosomes and MVs—were isolated from the urine specimens using a combination of centrifugation, ultracentrifugation, and treatment with DTT, as previously described [19]. A 50 mL exosome and MV pellets were resuspended in 100 μL of sterile PBS for protein quantification, electron microscopy and tunable resistive pulse sensing (TRPS), and the other 50 mL-pellet was resuspended in 100 μL of sterile PBS for RNA isolation. The exosomes and MVs were then immediately processed, as described below, to extract their RNA.

### Tunable resistive pulse sensing (TRPS)

A qNano Gold instrument (Izon Science Ltd., Christchurch, New Zealand) was used for TRPS. Both the EV preparations and the calibration particles (supplied by Izon) were diluted 1:100 in PBS. The exosomes were measured using NP150 nanopores and CPC200 calibration beads (mode 203 nm) while the MVs were quantified using NP400 and CPC400 beads (mode 335 nm); 40 µL of each EV sample type were loaded into the instrument and at least 500 events were measured for each sample. Data was recorded using the Izon Control Suite Software v.2.2.2.111. The default minimum blockade height (0.05 nA) for particle detection was used and pressure was set to 1.2 kPa.

### Transmission electron microscopy

Approximately 30 μL of each sample type (exosomes or MVs) was resuspended in PBS and placed on parafilm. A formvar carbon-coated nickel grid was then positioned on top of each drop for 15 min in dry environment before the samples were stained with 2% uranyl acetate for 5 min in the dark. Excess liquid was wicked off the grid, and grids were stored at room temperature until imaging was performed using a CM-10 Phillips transmission electron microscope.

### Western blot analysis

Total exosome and MV protein was prepared by resuspending each sample type in RIPA lysis buffer (50 mM Tris–HCl pH 7.4, 150 mM NaCl, 0.5% sodium deoxycholate, 1% NP-40, and 0.1% sodium dodecyl sulfate) containing 2 μg/mL leupeptin and 20 ng/mL aprotinin, on ice for 20 min. Total protein quantity was calculated by the Lowry method. Approximately 20 μg of total protein from each sample were electrophoresed on 4–12% Bis–Tris NUPAGE SDS-PAGE (Life Technologies, USA) under non-reducing conditions and was then transferred to a polyvinylidene fluoride (PVDF) membrane. These were subsequently blotted with primary antibodies against CD9 (Santa Cruz Biotechnology, USA), TSG101 (Abcam, UK), calnexin, GM-130, and nucleoporin 62 (Abcam, UK), and β-actin (Sigma, St Louis, USA) followed by a secondary IgG alkaline phosphatase peroxidase antibody (Sigma, St Louis, MO, USA). The immunoreactive bands were visualised using nitro blue tetrazolium/5-bromo-4-chloro-3-indolyl phosphate (NBT/BCIP, Sigma, USA) substrate system and quantified using TotalLab TL-100 (v.2008) software.

### RNA extraction

Total RNA was extracted from exosome and microvesicle pellets using a total exosome RNA and protein isolation kit (Invitrogen, Life Technologies, USA) from 100 μL of exosome suspension and stored at − 80 °C. The total RNA was quantified with a NanoDrop ND-1000 spectrophotometer (Thermo Fisher Scientific, USA), and the quality and size distribution of the samples was examined by capillary electrophoresis (Agilent 2100 Bioanalyzer, Agilent Technologies, Santa Clara, CA, USA) with a RNA 6000 Pico chip.

### Quantitative real-time reverse transcription PCR

For miRNA expression analysis, complementary DNA was synthesised from 5 μL of total RNA using a TaqMan™ MicroRNA Reverse Transcription kit (Applied Biosystems, USA). Briefly, 1.33 μL of cDNA was combined with TaqMan universal PCR master mix II, No UNG, and specific TaqMan microRNA assay primers for miR-146a (ID: 000468) and miR-335* (ID: 002185). The target microRNA data were normalised across the samples using a median normalisation procedure [19], and a synthetic *Caenorhabditis elegans* miR-39 (cel-miR-39) RNA oligonucleotide was spiked into the RNA samples as an internal control (ID: 000200).

All the reactions were run in triplicate, including the blank/negative controls without cDNA, on a LightCycler 480 II real-time PCR system (Roche) under following conditions: 95 °C for 10 min, 40 cycles of 95 °C for 15 s and 60 °C for 1 min; the data were analysed with LightCycler 480 software (v.1.5). Changes in gene expression were calculated using the ΔΔC_T_ method as follows: ΔC_T_ = C_T_ (target miRNA) − [(Average_cel-miR-39_Ct value of the given sample) − (Median_cel-miR-39_Ct value)]; ΔΔC_T_ = ΔC_T_ (exosome miRNA) − ΔC_T_ (MV miRNA); the fold change = 2^−ΔΔCT^. When we compared the influence of UAE, changes were calculated as: ΔC_T_ = C_T_ (target miRNA) − [(Average_cel-miR-39_Ct value of the given sample) − (Median_cel-miR-39_Ct value)]; ΔΔC_T_ = ΔC_T_ (MALB exosome or MV miRNA) − ΔC_T_ (NO MALB exosome or MV miRNA); the fold change = 2^−ΔΔCT^.

### Statistical analysis

Statistical analyses were completed using GraphPad Prism software v.6 (GraphPad Software, Inc. La Jolla, CA, USA) and SPSS software (SPSS, Chicago, IL, USA). To compare EV subgroups, the exosome miRNA data were presented as the mean fold change relative to the MV group, and to compare the effect of the presence or absence of UAE on miRNA levels, relative to the normal UAE. Mann–Whitney U tests were used to determine the significant differences in miRNA expression in EV subpopulations and the influence of UAE on EV miRNA expression. The association between miR-146a levels and UAE in exosomes and MVs was analysed by Spearman’s correlation coefficient. Receiver operating characteristic (ROC) curves were constructed using the expression value of each miRNA, and the area under the curve (AUC) and 95% confidence interval (CI) was calculated for each ROC curve. A p value < 0.05 was considered statistically significant.

## Results

### General characteristics of the study population

Results were obtained from 52 patients with essential hypertension (mean age = 53.7 ± 7 years, 65% male) in which 24 had increased albuminuria (UAE 162.78 ± 168.21) and 28 were normalbuminuric (UAE 3.36/2.28 mg/g). The main characteristics of the study population are shown in Table 1.

**Table 1:** Clinical characteristics of the patient groups

Variables	Albuminuric	No albuminuric
	(n = 24)	(n = 28)
Age (years)	52.6 ± 8.4	54.6 ± 5.6
Gender (male)	66.7%	64.3%
Systolic blood pressure (mmHg)	136 ± 11	136 ± 24
Diastolic blood pressure (mmHg)	84 ± 15	88 ± 15
Pulse pressure (mmHg)	52 ± 12	48 ± 16
Glucose (mg/dL)	119 ± 45	118 ± 40
Glycated haemoglobin (%)	6.6 ± 0.3	6.0 ± 0.8
Total cholesterol (mg/dL)	202 ± 34**	174 ± 28
LDL (mg/dL)	131 ± 30**	108 ± 25
HDL (mg/dL)	51 ± 14	49 ± 11
Triglycerides (mg/dL)	148 ± 76	129 ± 59
Plasma creatinine (mg/dL)	0.86 ± 0.06	0.89 ± 0.21
Glomerular filtration rate (mL/min/1.73 m^2^)	97 ± 27	88 ± 19
Waist (cm)	107 ± 15	99 ± 12
Central obesity (%)	54	50
Body mass index (kg/m^2^)	32 ± 7	30 ± 6
Obesity (%)	50	39.3
Obesity grade (%)		
Grade I	29	18
Grade II	8	11
Grade III	13	11
Diabetes (%)	38	32
Dyslipidemia (%)	88	82
Smoking (%)	46	46
Ex-smoking (%)	29	14
Urinary albumin excretion/creatinine (mg/g)	162.8 ± 168.2***	3.3 ± 2.3
Antihypertensive treatment (%)		
ACEi	100	100
ARB	93	92
CCB	64	38
Diuretics	64	63

*ACEi* angiotensin converting enzyme inhibitors, *ARB* angiotensin II receptor antagonists, *CCB* calcium channel blocker, *HDL* high-density lipoprotein, *LDL* high-density lipoprotein

The data are expressed as the mean ± SD, unless noted otherwise. The glomerular filtration rates were calculated using the MDRD formula. Comparisons between microalbuminuric groups: **p < 0.01, ***p < 0.001

### Isolation and characterisation of urinary extracellular vesicles

**Fig. 1:**
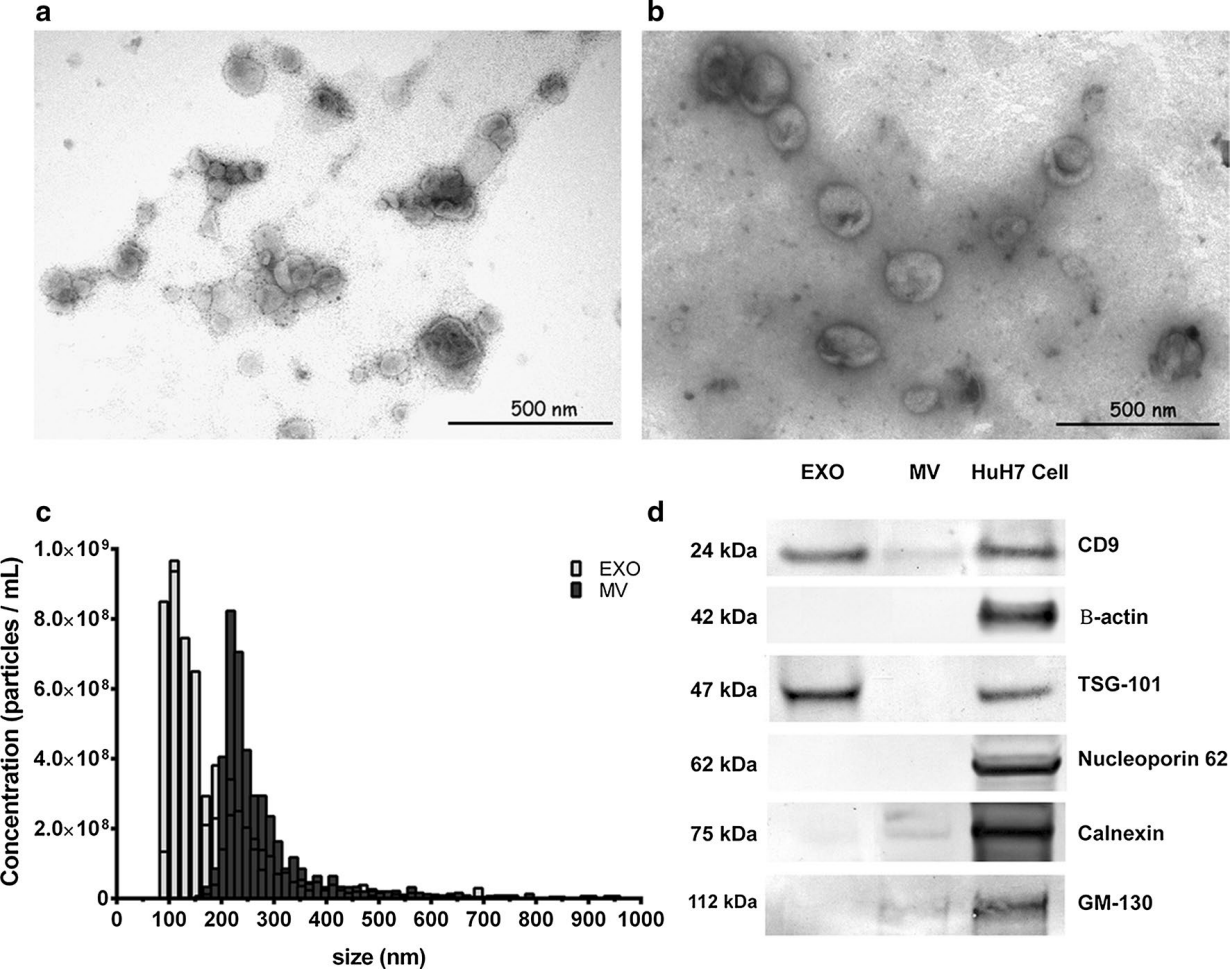
Characterisation of urinary exosomes isolated by ultracentrifugation. Transmission electron microscopy (TEM) micrographs of extracellular vesicle isolations stained with uranyl acetate in **a** exosomes and **b** microvesicles, bar represents 500 μm. **c** The size distribution of urinary exosomes and microvesicles by tunable resistive pulse sensing analysis of 500 events per sample (n = 4). **d** Western immunoblotting of exosomes and microvesicles isolated from urine using exosomal markers, including Tsg101 and CD9, and non-exosomal markers including calnexin, nucleoporin, p62, GM-130 and β-actin. Whole-cell lysates from HuH7 cells were loaded as a positive control

To validate the EV purification protocol, we analysed the morphology of the urinary MVs and exosomes by transmission electron microscopy (Fig. 1a, b). The isolated EVs were then subjected to TRPS (Fig. 1c) which clearly showed the isolation of two separate EV subpopulations. In addition, the exosome fraction exclusively expressed TSG-10, and the isolated EVs generally expressed CD9, especially in the exosomes. These are well-established surface and cytoplasmic markers for exosomes, which were also evident in Western blot analysis (Fig. 1d). Moreover, non-exosomal markers were highly enriched in the positive control cell pellet (HuH7), were only slightly present in the MV fraction in the case of calnexin and GM-130, and were absent in the exosome-enriched fraction.

### microRNA-146a and miR-335* levels in extracellular vesicle fractions

**Fig. 2:**
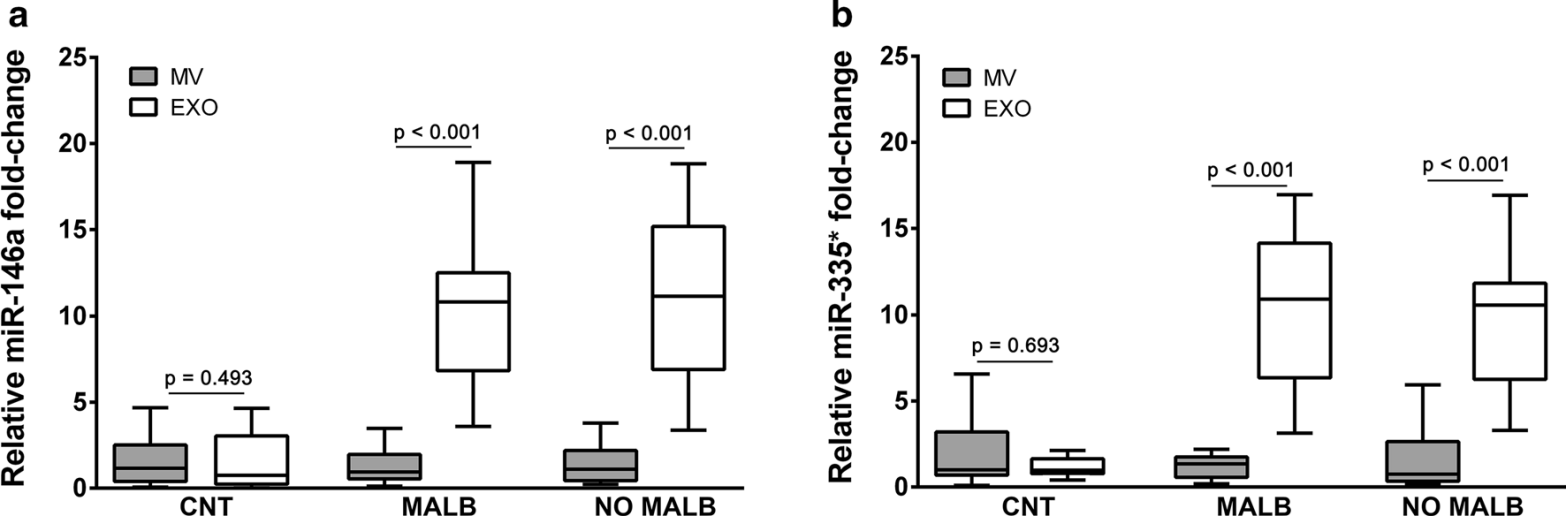
Box plots of the miR-146a and miR-335* levels in exosomes and microvesicles **a** in albuminuric (MALB) patients and **b** in non-albuminuric (NO MALB) hypertensive patients. The relative expression was calculated using the 2^−(ΔΔCt)^ method. The median Ct value of the spike-in cel-miR-39a in all the samples was used as a control. The data were compared using the Mann–Whitney U test. *EXO* exosomes, *MV* microvesicles

Quantitative RT-PCR indicated that EV miR-146a and miR-335* expression in the isolated urinary exosomes was significantly higher than in the MV subpopulation (12-fold increase, p < 0.001 in both cases) in hypertensive patients (data not shown). When we compared EV fractions in patients with or without increased albuminuria (MALB vs. NO MALB), miR-146a and miR-335 were present in high amounts in exosomes in both groups compared to the MV subpopulation (11-fold increase, p < 0.001 (Fig. 2a, b), although this difference was not significant when compared to the control group.

### Exosome and microvesicle miR-146a and miR-335* levels in hypertensive patients with or without albuminuria

**Fig. 3:**
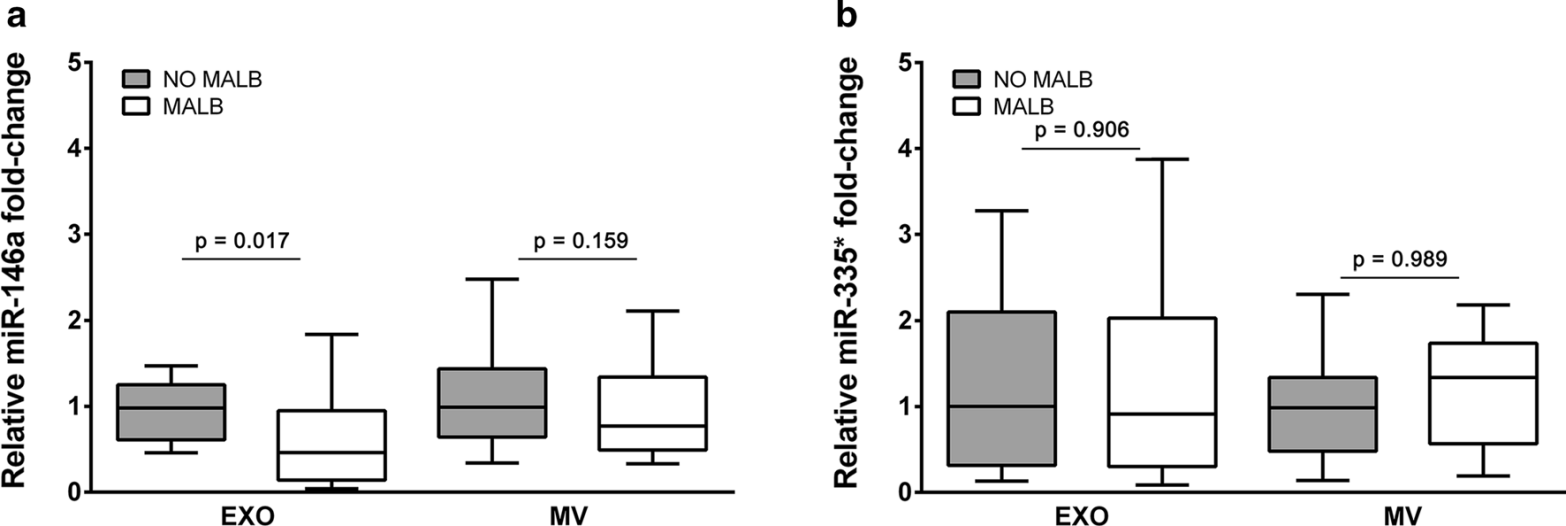
Box plots of the miR-146a and miR-335* levels in exosomes and microvesicles according to the presence of **a** albuminuria for miR-146a and **b** for miR-335*. Relative expression shown was calculated using the 2^−(ΔΔCt)^ method. The median Ct value of the spike-in cel-miR-39a in all samples was used as a control. The data were compared using the Mann–Whitney U test. *EXO* exosomes, *MV* microvesicles

Urinary exosome miR-146a was significantly lower in the albuminuric group compared to the non-albuminuric patients (2.5-fold less, p = 0.017; Fig. 3a). In comparison, miR-146a levels were slightly lower in the MV-enriched fraction in the albuminuric group, although this difference did not reach statistical significance (1.4-fold decrease, p = 0.159). Finally, miR-335* expression levels were similar in the presence or absence of albuminuria (Fig. 3b), thus supporting the specificity of the results we obtained for miR-146a.

### Exosome miR-146a expression and urinary albumin excretion in hypertensive patients

**Fig. 4:**
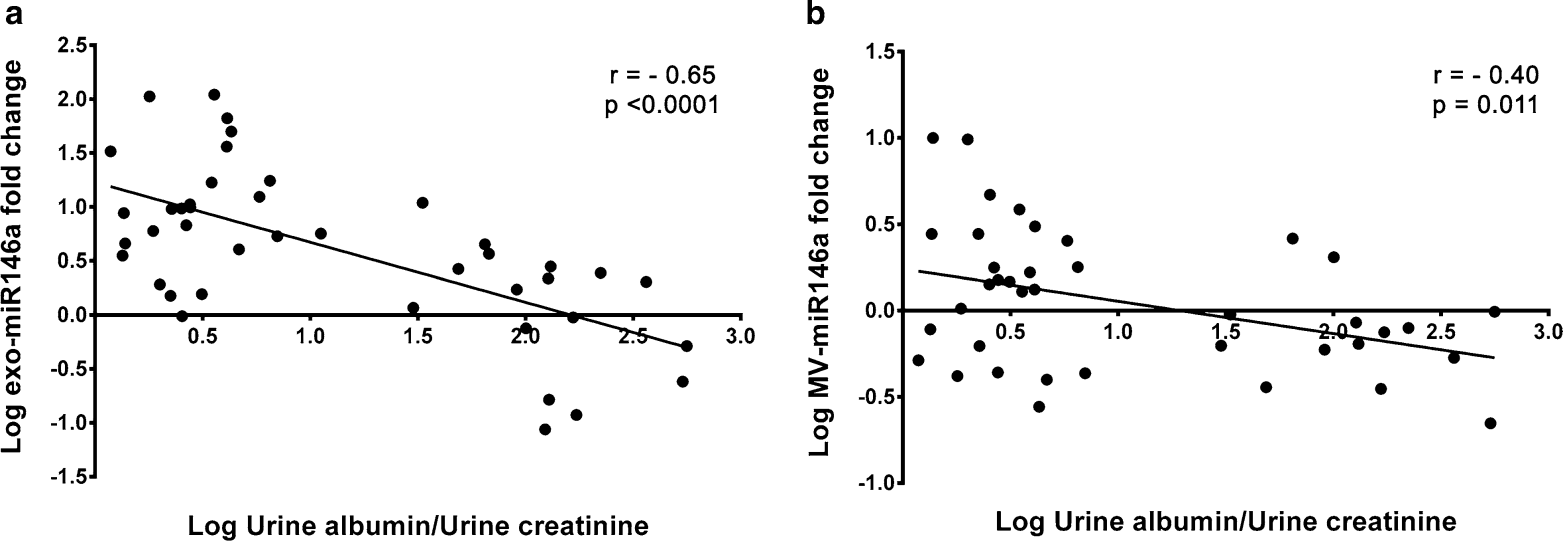
Associations between urinary miR-146a levels with urinary albumin excretion, normalised by urinary creatinine, in hypertensive patients. The data were assessed using the Spearman correlation coefficient. Inverse associations were found for exosomal (**a**) and microvesicle (**b**) miR-146a expression levels

**Fig. 5:**
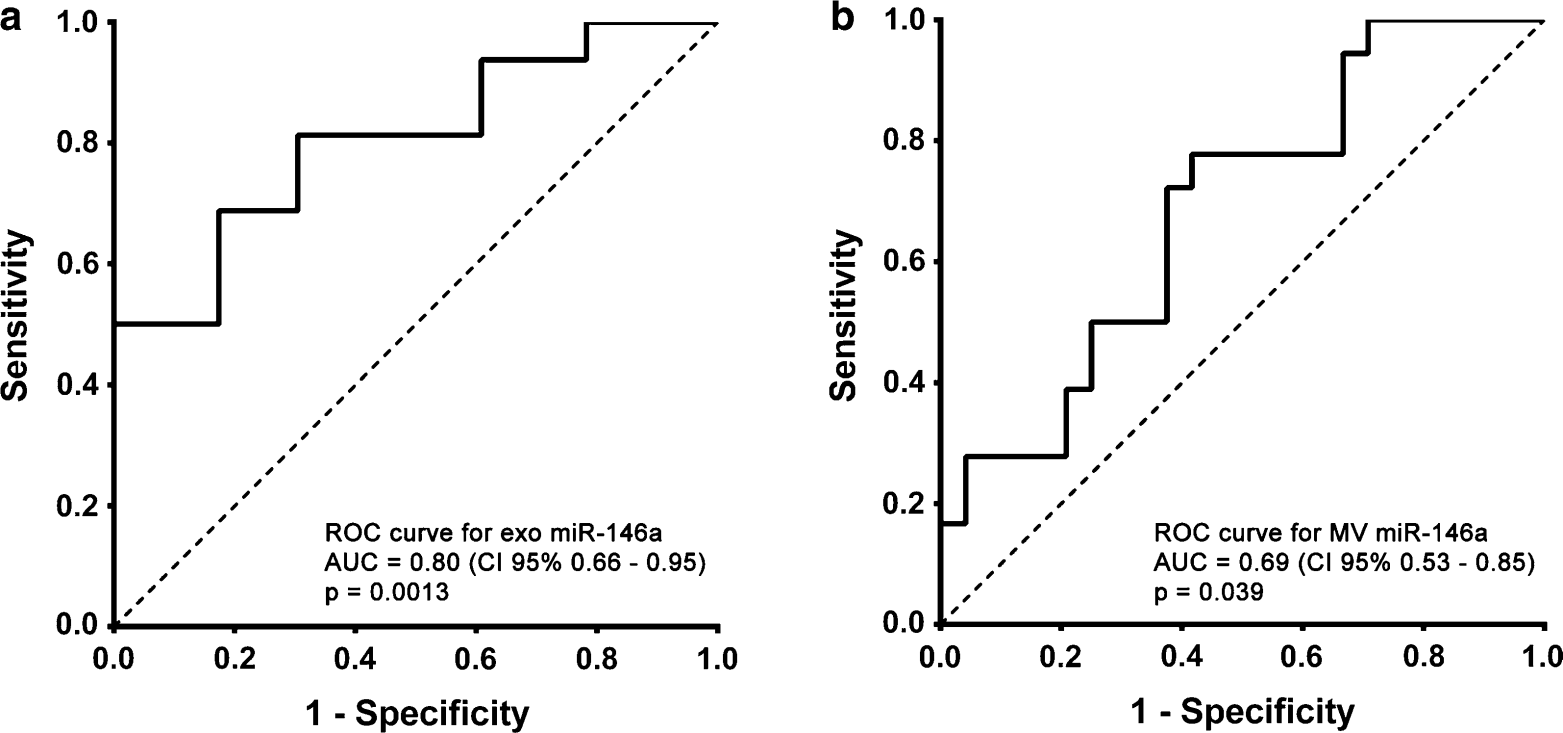
ROC analysis of urinary microRNA expression in extracellular vesicles from normalbuminuric and microalbuminuric hypertensive patients. Receiver operator characteristic (ROC) curves were constructed using the microRNA expression values for miR-146a in exosomes (**a**) and microvesicles (**b**). The area under the curve (AUC) and 95% CI were computed and are shown for each ROC curve. The Wilcoxon and Mann–Whitney U tests were used to test the null hypothesis that the AUC is equal to 0.5, and the probability values for each test are shown

Urinary exosome miR-146a expression was highly correlated with urinary excretion of albumin in all the hypertensive patients, as shown in Fig. 4 (log-transformed expression levels and UAE: r = −0.65, p < 0.001). In contrast, miR-146a was only slightly associated with UAE in the MV fraction (r = −0.40, p = 0.011) (Fig. 4). There was no correlation between UAE and miR-335* in MV or in exosome fractions. The ROC curves constructed for miR-146a in the exosome and MV fractions to compare their diagnostic value in predicting the presence of UAE (Fig. 5) showed an AUC of 0.80 for exosome miR-146a (95% CI 0.66, 0.95; p < 0.001) thus indicating the predictive power of this miRNA in urinary exosomes in these patients; the AUC for MV miR-146a was 0.69 (95% CI 0.53–0.85; p = 0.039). These data show that expression of miR-146a in exosomes could indicate the presence of albuminuria.

## Discussion

This study shows that miR-146a is highly enriched in urinary exosomes compared to the MV subpopulation in hypertensive patients. In addition, it is detected at significantly lower levels in urinary exosomal samples from patients with essential hypertension and albuminuria compared to normoalbuminuric hypertensive patients. However, there were no differences in the control group. EV subtype analysis, a relevant feature of the present study, further indicated that urinary exosomal miR-146a is a good indicator of incipient renal injury and could be used as a potential biomarker for the early detection of renal injury. Recognition of the importance of UAE as a prognostic marker in hypertension is based on cross-sectional studies, which demonstrate clustering of cardiovascular risk factors and organ damage associated with an increase in UAE [4, 6]. However, the value of a single UAE measurement as a risk marker for the development of chronic kidney disease or end stage renal disease remains controversial. The reason for this discrepancy may be the potential mechanisms involved in increasing UAE: either via a predominance mechanism through glomerular leakage, or because of a reduction in the tubular capacity of uptake and metabolization [25].

An association between circulating and tissue miRNA expression and renal injury has been described in various diseases including diabetic nephropathy, lupus nephritis, IgA nephropathy, and focal segmental glomerulosclerosis [26–28]. In addition, recent studies have also shown that miRNA profiles are potential indicators of renal damage in hypertension. However, most of these studies analysed plasma and tissue miRNA expression profiles [29–31] but, to our knowledge, none have focussed exclusively on the potential of urinary exosome miRNAs as biomarkers for early renal injury in hypertension. Our results indicate that urinary exosomal miR-146a levels are significantly lower in albuminuric patients compared to normoalbuminuric patients. This observation fits generally with previous reports showing that low glomerular levels of miR-146a had a high ACR at the time of biopsy in diabetic patients [23]. In addition, UAE significantly negatively correlated with urinary exosomal miR-146a expression in our hypertensive patients. These findings also suggest that exosomal miR-146a levels could be used as a potential indicator of renal injury. Furthermore, our ROC curve analysis indicates that urinary exosome miR-146a levels can discriminate the presence of microalbuminuria, although the sensitivity and specificity of this discrimination could perhaps be improved in a study with a larger cohort.

Of note, the miRNA types contained in MVs and exosomes were significantly different: in this case miR-146a and miR-335* were significantly higher in exosomes compared to MVs. This suggests that miRNAs are selectively sorted into exosomes rather than MVs, as also shown in previous reports [32, 33]. Moreover, miR-146a levels also differed according to the presence of UAE, indicating that this sorting may depend on the physiological context [34]. These data suggest that miR-146a loading into exosomes in hypertensive patients is not a passive process and that this sorting may be based on biological function.

Previous evidence seems to suggest that miRNA-146a is loaded into exosomes as a biological response to the different renal pathological processes that occur during hypertension (endothelial inflammation, fibrosis, podocyte damage, etc.) [9, 20, 21]. Furthermore, it was also recently reported that miR-146a was augmented in lupus nephritis [19, 22] and that is highly expressed in podocytes [23, 24]. Therefore, urinary exosomal miR-146a levels could be quantified as a potential indicator of early-stage renal injury in these patients. Low levels of exosomal miR-146a in hypertensive patients with albuminuria are likely to promote this pathology and contributes to advancing renal injury. However, further studies looking at a much wider range of miRNAs in different EV subpopulations would be required to further support the finding that these miRNAs are enriched in exosomes relative to MVs in hypertensive patients.

## Conclusion

This is the first study to report high levels of miRNA enrichment in urinary exosomes compared to MVs in hypertensive patients and that urinary exosome levels of miR-146a are significantly associated with UAE and are decreased in patients with albuminuria. These findings lead us to conclude that urinary exosome miR-146a may serve as non-invasive biomarker for early renal injury in hypertension, such as albuminuria. As early diagnosis remains the key to improving renal damage, further studies are warranted to firmly establish urinary exosome miRNA signatures indicative of renal damage in hypertension.

## Abbreviations

ACR: albumin creatinine ratio
AUC: area under curve
cDNA: complementary DNA
EV: extracellular vesicle
MALB: microalbuminuria
MiRNA: micro RNA
mRNA: messenger RNA
MV: microvesicles
ROC: receiver operating curve
RT-PCR: real time PCR
TRPS: tunable resistive pulse sensing
UAE: urinary albumin excretion
